# Role of anti-*Giardia* recombinant cyst wall protein IgG polyclonal antibodies in diagnosis and protection

**DOI:** 10.1186/s13568-022-01484-w

**Published:** 2022-11-24

**Authors:** Ahmed Maher, Donia Atallah, Mahmoud Hassan, Mariam Hammad, Mohaned Galal, Saif-Eldin Mohamed, Yara Abdelkafy, Alyaa Farid

**Affiliations:** 1 Biotechnology/Biomolecular Chemistry Program, Faculty of Science, Cairo University, Cairo, Egypt; 2 Immunology Division, Zoology Department, Faculty of Science, Cairo University, Cairo, Egypt; 3 Biotechnology Department, Faculty of Science, Cairo University, Cairo, Egypt

**Keywords:** *G. duodenalis*, Sandwich ELISA, IgG, pAb

## Abstract

*Giardia duodenalis* (*G. duodenalis*) is an infectious protozoan that has a global distribution especially in the hot climate. Around 200 million people are infected worldwide annually by *Giardia*, but infection is not always accompanied by symptoms, especially in endemic countries. Using traditional microscopy techniques in diagnosis, both in stool and water samples were less sensitive when compared to immunological methods; and the need for new diagnostic methods was necessary. Also, protection from infection is required in endemic areas. Therefore, the study aimed to produce anti- *G. duodenalis* IgG polyclonal antibodies (pAbs) by immunizing rabbit by *G. duodenalis* cyst recombinant protein. The produced antibodies were evaluated in the detection of *G. duodenalis* antigens in patients’ stool and water samples from endemic areas across River Nile; where pAbs were used as a coating and a peroxidase conjugate antibody in sandwich ELISA. Moreover, pAbs were tested for the protection of mice from giardiasis. Sandwich ELISA using pAb has succeeded in the detection of *G. duodenalis* coproantigens in stool samples by a sensitivity of 97% and a specificity of 92.72%. Moreover, *G. duodenalis* cyst was detected in only seven water samples by ordinary microscopy; while sandwich ELISA revealed nineteen positive results. IgG pAb (1/200 µg/ml) protected mice from giardiasis; which was evident from the reduction in cysts and trophozoites numbers. We recommended the use of sandwich ELISA to monitor water quality, investigate environmental contamination and diagnosis in patients' stools. The pAbs can be prepared in large amount and used in field diagnosis and protection. This will help in the early diagnosis of *G. duodenalis* in water, which in turn can control outbreaks in rural areas.

## Keypoints

1. IgG pAbs detected G. duodenalis antigens in stool and water samples.
2. IgG pAbs protected mice from giardiasis.
3. IgG pAbs can be produced in large quantities.

## Introduction

*Giardia* (*G.*) *duodenalis*, commonly named *G. lamblia* or *G. intestinalis*, is a parasitic protozoan that parasitizes the small intestine of a variety of mammals causing giardiasis (Lalle and Hanevik, 2018). Giardiasis is a disease that induces fatigue, soft stool, abdominal cramps, vomiting and dehydration (Lalle and Fiorillo, 2019); the severity of infection depends on the age and immune conditions of the infected host (Leung et al. 2019). Giardiasis is a self-limiting illness and most often, infections are asymptomatic. In the developing countries, malnourished children are the most affected; also, immunocompromised individuals are at a risk of severe acute or chronic infection by *G. duodenalis*; in addition, being a daycare worker is a risk for infection due to close contact with children (Thompson et al. 1993; Thompson, 2000). Outbreaks are widespread in places with poor water treatment, particularly in a developing country, in which the rates of infection might reach fifty percent of the overall population. Infection was linked to inadequate sanitation, lower water quality, and overpopulation in low-income areas (Younas et al. 2008).

The precise giardiasis detection is critical for the management of disease and therapy; and there are several approaches for a diagnosis like the traditional microscopic methods, immunological and molecular techniques. The microscopy method depends on the detection of cysts and/or trophozoites in the patient's saline preserved stool (Fink and Singer, 2017). The examination of the stool sample is the most common, cheapest, and most straightforward screening test (Khan, 2001). The microscopic examination method offers low sensitivity due to the excretion of the irregular cysts, the possibility of confusion with other infections, and ghost cysts (empty cysts that are often not distinguished). Furthermore, its sensitivity is affected by the infection severity, the stool sample freshness, the stain, and the microscopist’s level of experience (Pawlowski et al. 2009). Though it is the greatest common approach, it is not extremely antigen sensitive compared to the immunological approaches that show high sensitivity. In addition, the cysts can be degraded in not well-preserved stool samples (Hooshyar et al. 2019).

In developing countries, detection of *G. duodenalis* either in the stool or environmental samples has been accomplished with antigen immunodetection techniques (Christy et al. 2012). The antigen immunoassays have been preferred in routine diagnostic laboratories due to its high sensitivity compared with traditional microscopic-based techniques (Alexander et al. 2013). Moreover, antigen detection techniques required much less time allowing the screening of large numbers of samples in a short time with high specificity and sensitivity (Nooshadokht et al. 2017).

Also, detection of the cysts in samples of water relied upon the direct examination of water samples. Hall and Glysson (1991) used membrane filtration and percoll-sucrose flotation for cysts collection from sewage raw water samples and cyst examination by epifluorescent microscope. The United State Environmental Protection Agency (U.S. EPA) in the period 1996 to 1999 has introduced standard protocols for the detection of *Giardia* cysts and/or oocysts of *Cryptosporidium parvum* in water (U.S. Environmental Protection Agency, 2001a, U.S. Environmental Protection Agency, 2001b). These protocols (method 1622 and 1623) are based on a scheme involving isolation of parasites from the water, their marking and counting using fluorescence microscopy. Later on, McCuin and Clancy (2003) showed the disadvantages of the U.S. EPA 1623 method which are expensive, time-consuming and only trained and competent analysts can perform the analysis.

The diagnosis through immunological techniques is accomplished either by detecting antibodies (IgG and/or IgM) or antigens by enzyme-linked immunosorbent assay (ELISA), immunofluorescence assay (IFA), and western blot (Gutiérrez-Cisneros et al. 2011; Júlio et al. 2012). However, one of the drawbacks of antibodies screening test is the probability of a false-positive result due to: 1- IgG remains in the serum for approximately eighteen months after infection, 2- levels of IgM decline after two or three weeks after infection because IgM is regarded as a useful detector in acute infection (Heyworth, 2014) and 3- Cross-reactivity against other protozoan antigens (Pacheco et al. 2020). Researchers reported that 11.6% and 24.4% of the patients (infected *with Endolimax nana* *or Escherichia coli,* respectively) showed high reactivity for anti-*G. duodenalis* IgA and IgG; they explained this cross-reactivity with the similarity of protozoans’ antigens and immunological memory due to the previous giardiasis. Ricciardi and Ndao (2015) recommended the use of recombinant antigens instead of crude antigens to offer high specificity. Antigen detection techniques are considerably superior to antibodies detection assays because it is more sensitive and offers a precise diagnosis (Al Saeed and Issa, 2010).

On the other hand, secretory antibodies IgA (sIgA) plays an important role in the clearance of *G. duodenalis* from the intestinal tract (Faubert, 2000). Various researches imply that B lymphocytes play a significant role in eradicating *G. doudenalis* infection. For instance, when humans are infected with *G. duodenalis*, anti-*Giardia* antibodies (IgA, IgM, and IgG isotypes) are produced in the sera and gastrointestinal secretions, and the production of these particular antibodies was correlated with the clearance of the parasite (Snider and Underdown, 1986; Nash et al. 1987; Daniels and Belosevic, 1994; Faubert, 2000). Heyworth (1986) reported that anti-giardial IgA and IgG antibodies covered trophozoites in infected mice. Chronic giardiasis infection is more common in people with immunological abnormalities that primarily impact B lymphocytes, like X-linked agammaglobulinemia (Van der Hilst et al*.* 2002). A comparable mutation in mice causes the primary *Giardia* infection to last longer (Snider et al. 1988). Klotz and Aebischer (2015) concluded that antibodies increased effector pathways were associated with symptomatic giardiasis infections and may even be able to regulate them. Due to the presence of *G. duodenalis*-specific sIgA in the mother’s breast milk, epidemiological studies have linked nursing to protect against symptomatic giardiasis in newborn children (Tellez et al. 2005; Ignatius et al. 2012). Selim et al. (2017) produced anti- *Giardia* polyclonal antibodies (pAbs) in chicken egg yolk and investigated its efficiency in *Giardia*-infected mice. They reported the enhancement in the mice body weight and the reduction in cysts shedding; moreover, these antibodies decreased the attachment of *Giardia* to Int-407 cells. Yokoyama et al. (2007) and Ibrahim et al. (2008) reported that passive immunization had a therapeutic value against many infections in humans and animals. Marcotte and Hammarström (2015) added that although passive immunity is temporary, it is immediate and lasts from a few weeks to4 months. DuBourdieu (2019) reported that passive immunity is a novel strategy for delivery protection against pathogens because of the rise of drug-resistant pathogens and immunocompromised individuals who are unable to respond to conventional vaccines.

From this point, our study aimed to produce anti- *G. duodenalis* IgG pAbs by immunizing New Zealand white rabbit by *Giardia* cyst Recombinant protein. The produced antibodies were evaluated in the detection of *G. duodenalis* antigens in the stool samples of infected patients and water samples from endemic areas across River Nile; where pAbs were used as a coating and a peroxidase conjugate antibody in sandwich ELISA. Moreover, the hyperimmune sera (pAbs) was used in the protection of mice against giardiasis.

## Materials and methods

### Production of anti-*G. duodenalis* IgG pAbs

Three kilograms weighted two rabbits were immunized with *Giardia* cyst recombinant protein according to Tendler et al. (1991). Before the immunization, each rabbit was tested to ensure that it is clean from any parasitic infection; and kept in the animal house in Cairo University under standard laboratory conditions at 21 °C, 15% moisture, drinking filtered water, diet (15% protein, 3% fat and 22% fibers). The experimental procedures were approved by the Institutional Animal Care and Use Committee (IACUC) of Cairo University. *Giardia* cyst recombinant protein (MBS318900) was purchased from MyBioSource (USA). Briefly, primers were used to amplify a part of the gene cell wall protein 2 (cwp2) (Lee and Faubert, 2006). This protein is expressed in *Pichia pastoris* and significantly increases the protein production yields. Protein was purified by affinity chromatography and was dialysed against PBS (pH 8.0). Each rabbit was immunized with the following: 1—an intramuscular injection of the priming dosage [one mg *Giardia* cyst recombinant protein (MBS318900, MyBioSource, USA) mixed 1:1 with complete Freund's adjuvant (Sigma)], 2—two booster doses of 0.5 mg *Giardia* cyst recombinant protein emulsified in incomplete Freund's adjuvant, 2 weeks following the priming dose, and 1 week after the first booster. Blood samples were collected from the animals at each injection (boosting) to measure the titer of produced pAbs. After one month, the animals were sacrificed for the collection of blood samples. Serum was fractionated and stored at − 20 °C till used. The level of pAbs was elevated one week after the 1st booster dose; and 3 days after the 2nd booster dose, rabbits’ sera gave a high titer against G. *duodenalis* cyst recombinant protein with the optical density of 3.2 at 1/100 dilution.

### Purification of rabbit anti- *G. duodenalis* IgG pAbs

Ammonium sulphate precipitation (Nowotny, 1979) and caprylic acid purification (Nowotny, 1979) were used to purify the animal sera (Mckinney and Parkinson, 1987). Purified antibodies were tested for protein content (Bradford, 1976), and molecular weight was calculated using a 12.5 percent SDS-PAGE under reducing conditions (Myers, 1995; Thaumaturgo et al. 2002). Indirect ELISA was used to test the reactivity of pure IgG pAbs. According to Tijssen and Kurstak, IgG pAbs were tagged with Horseradish Peroxidase (HRP) (Periodate Method) (Tijssen and Kurstak, 1984). Crude rabbit serum with anti-*G. duodenalis* antibody had a total protein concentration of 9.2 mg/ml. Protein content was reduced to 3.5 mg/ml after purification. IgG pAbs were represented as heavy and light chain bands at 53 and 31 kDa, respectively, in a 12.5 percent SDS-PAGE under reducing conditions.

### Application of pAbs in diagnosis

#### Study population

The study was conducted in endemic areas with giardiasis (Sharkia and Ismailia governorates) in the period from March 2021 to January 2022 on 100 patients infected with giardiasis and 30 patients infected with other parasites (*Cryptosporidium, Entamoeba (E.)histolytica* and *Escherichia (E.) coli*). Prior to sample collection, a personal interview with each patient was held in order to make informed consent. In addition, 25 healthy volunteers' served as the parasite-free-healthy negative control. All fecal samples were diagnosed with merthiolate-iodine-formaldehyde concentration technique (MIFC) according to Blagg et al. (1995).

### Water samples collection and concentration

Collection of water sample and concentration of parasite was accomplished by membrane filtration (U.S. Environmental Protection Agency, 2001a). Twenty water samples were collected randomly from different endemic areas of River Nile, where patients of positive *Giardiasis* were residing. Half of the water samples were collected from rural areas and the other half from urban areas. Water samples were collected in sterilized and clean-coded 10 L (L) carboys, labeled with date and place of collection. In addition, 20 distilled water samples served as a negative control. *Giardia* cysts were separated from debris by Percoll-sucrose flotation (U.S. Environmental Protection Agency, 1995).

The membrane filtration method, using a polycarbonate membrane filter (0.1 μm pore size) was used to concentrate water samples (U.S. Environmental Protection Agency, 2001a). Several filters were utilized for each sample of highly turbid water. The sediment was washed with 10–50 ml eluting solution containing Na dodecyl sulfate (1%), tween eighty (1%) and phosphate-buffered saline. Samples were centrifuged for 20 minutes at 3000 rpm for cysts concentration in 50 ml Falcon tubes. The concentrated samples were kept refrigerated in an equivalent amount of formalin (5%) until flotation on Percoll-sucrose gradients and examination under a microscope. Processed water samples were examined directly by light microscopy using Lugol's iodine solution, for detection of *Giardia* and amebas of the *E. histolytica/dispar/moshkovskii* complex cysts. *Cryptosporidium* oocysts was detected with the U.S. Environmental Protection Agency method 1623 (Sinclair, 2000). Where, oocysts concentration was accomplished by filtration; followed by immunomagnetic separation (U.S. Environmental Protection Agency, 2001a), staining by modified Ziehl–Neelsen technique and then microscopic examination. Briefly, samples were air-dried and ethanol fixed. Slides were soaked with alkaline fushin, heated for 5 minutes, then washed with water, treated with H_2_SO_4_ (205%), and then counter stained for 1 minute with methylene blue (1%) (Smith et al. 1989; Farid et al. 2021).

### Detection of *G. duodenalis* antigen in stool and in water samples by sandwich ELISA

Stool samples were prepared for ELISA by mixing one part of a fresh stool sample with three parts of PBS/tween 20 followed by centrifugation for five minutes at 900 xg; the supernatant was stored at − 80 °C until use. The purified IgG pAb was used for the detection of *G. duodenalis* cyst’s antigens in both stool and water samples; it was employed as an antigen-capture and HRP antibody conjugate in sandwich ELISA. After standardization of sandwich ELISA, the selected concentration of coating and HRP conjugated antibody was 1/100 and 1/50 µg/ml, respectively. The plates were coated with 100 μl pAb (1/100) per well and incubated at room temperature overnight; followed by washing three times in PBS/Tween (0.1 M and pH 7.4). Plates were blocked by incubating, for two hours at room temperature, with 200 μl per well of BSA/PBS/T (0.1%) followed by three times washing. 100 μl of either processed stool or water sample were pipetted into the wells in duplicate and incubated at room temperature for 2 hours followed by washing three times. 100 μl per well of peroxidase-conjugated PAb (1/50) was added and incubated in room temperature for one hour. Each well received 100 μl of the substrate (o-Phenylenediamine), and the plate was incubated for half an hour at room temperature in the dark; the reaction was then stopped with the addition of 50 μl/well of H_2_S0_4_. The absorbance was measured at 492 nm using an ELISA reader.

### Application of IgG pAbs in protection against *G. duodenalis* infection

#### Experimental design

Twenty male mice (8 weeks old) were purchased from Theodore Bilharz Research Institute (TBRI) and maintained in the animal house, Faculty of Science, Cairo University. Mice were divided into four groups (five/group): healthy control mice (group I), *G. duodenalis* infected unprotected mice (group II), *G. duodenalis* infected protected mice that received an intraperitoneal injection with 100 μl of purified pAb with a concentration of 1/100 (group III) or 1/200 diluted in PBS (group IV). Three days after pAb administration, mice were orally infected by 10^4^ *G. duodenalis* cysts (obtained from the fresh stool of heavily infected mice) suspended in one ml of PBS. Three days post-infection, fresh stool samples were examined by the MIFC technique (Blagg et al. 1955). Ten days post-infection, animals were anesthetized with 80 mg/kg of pentobarbital. Blood was allowed to clot and serum samples were divided into aliquots and preserved at − 80 °C. Intestinal segments (2 cm) were collected, from all experimental groups, and examined to count the number of trophozoites.

### Histopathological examination

Tissues were fixed for 24 h in buffered formalin (10%). Following fixation, samples were rinsed with tap water, dehydrated in ascending concentrations of ethanol (70, 90, 95, and 100%), cleared in xylene, and then embedded in paraffin wax at 55 °C. Each tissue was divided into five slices of 4 m thickness, which were then stained with hematoxylin and eosin. Slides were first placed in xylene to melt the wax, followed by 2 minutes in pure alcohol, 1 minute in 95% alcohol, and one minute in 70% alcohol. Slides were stained for 5 minutes with hematoxylin and then rinsed in tap water. A small amount of excess stain was cleaned using alcohol. Sections were stained with eosin (4–5 min), then rinsed in water, 95% alcohol and xylene (Farid et al. 2022a, Farid et al. 2022b).

### Cytokines measurements

Interferon (IFN)-γ, interleukin (IL)-4 and IL-6 were measured in serum samples by mouse ELISA kits (ab100689, ab100710 and ab222503, respectively; abcam, USA) according to the manufacturer’s instructions.

### Statistical analysis

The data of sandwich ELISA were displayed as mean (X) ± standard deviation (SD); and the Cut-off values = mean value of healthy volunteers + 2 (SD). The percentages of sensitivity, specificity, positive predictive value (PPV) and negative predictive value (NPV) were calculated for each test. Analysis of cytokines results were performed by SPSS for Windows (version 11). Results were expressed as mean ± SD and significance of differences were examined by Student's t-test; where, the result was considered significant when P < 0.05 (Farid et al. 2022c).

## Results

### Microscopic examination of stool samples

According to the microscopic examination of stool samples, 91 patients from 100 *G. duodenalis* infected patients groups were positive with *G. duodenalis* cyst where 9 patients gave false-negative results.

### Microscopic examination of water samples

From microscopic identification of protozoa stages in water samples, *G. duodenalis* cyst was found in 11 water samples. According to cyst load, 25% of water samples carry moderate cyst load and 30% have a light cyst load. 15% of moderate cyst load was localized in water samples from rural areas. On the other hand, 10% of the light load was in urban areas (Table 1).

**Table 1:** Number of water samples contaminated by *G. duodenalis* cyst and cysts intensity

Water sample (20 sample)	Rural areas		Urban areas		Total	
	No.	%	No.	%	No.	%
Light cyst load (1–2 cyst/slide)	4	20	2	10	6	30
Moderate cyst load (3–4 cyst/slide)	3	15	2	10	5	25

### Detection of *G. duodenalis* cyst antigens in the stool samples of infected patients

The *G. duodenalis* infected groups achieved a mean optical density (OD) value (0.72) that was higher than the cut-off value (0.23); and higher than those of the healthy control group (0.21) and other parasites infected groups (0.11, 0.21 and 0.23 for *Cryptosporidium*, *E. histolytica* and *E. coli*, respectively) (Table 2). Three cases out of *G. duodenalis* infected samples showed false-negative results to record sensitivity of 97%. All the twenty-five negative controls showed OD values lower than the cut-off value; while four out of other parasites groups showed false-positive results recording 92.72% specificity, 96.03% PPV and 94.44% NPV (Table 3).

**Table 2:** Detection of *G. duodenalis* cyst antigens in stool samples by sandwich ELISA

Groups	Positive cases		Negative cases	
	*X* ± *SD*	No.	*X* ± *SD*	No.
Healthy volunteers (25)			0.21 ± 0.01	25
*G. duodenalis* infected patients (100)	0.72 ± 0.01	97	0.25 ± 0.05	3^a^
*Cryptosporidium* (10)	0.11 ± 0.11	2^b^	0.21 ± 0.14	8
*E. histolytica* (10)	0.21 ± 0.16	1^b^	0.12 ± 0.03	9
*E. coli* (10)	0.23 ± 0.01	1^b^	0.14 ± 0.03	9

X: mean OD reading at 492 nm

^a^False − ve results

^b^False + ve results

*SD* standard deviation

**Table 3:** The specificity, sensitivity, NPV and PPV of microscopic examination and sandwich ELISA in stool samples

Key features	Direct smear %	Sandwich ELISA %
Sensitivity	91	97
Specificity	100	92.72
PPV	100	96.03
NPV	85.93	94.44

### Detection of *G. duodenalis* cyst antigens in water samples

After microscopic analysis, the collected water samples (n = 20) were classified into two groups: three water samples contaminated with other protozoa (*cryptosporidium* n = 1 and *E. histolytica* n = 2), seven water samples with *G. duodenalis* cyst. Sandwich ELISA based on these results in order to evaluate the efficacy of prepared purified IgG pAbs in the diagnosis of *G. duodenalis* cyst in water samples. According to the cut-off valve (0.263); 19 water samples gave positive results, and all true negative control samples were below the cut-off value. Thus ELISA proved to be much more sensitive than microscopy techniques by detecting *G. duodenalis* cyst antigens in 19 water samples (Table 4).

**Table 4:** Detection of *G. duodenalis* cyst antigens in water samples by sandwich ELISA

Water samples	Positive		Negative	
	*X* ± *SD*	No.	*X* ± *SD*	No.
True negative controls			0.163 ± 0.05	20
Tested water samples (n = 20)				
Nil			0.271 ± 0.03	1
Light cyst load:	0.534 ± 0.06	7		
Moderate cyst load	0.682 ± 0.15	12		

X: mean OD reading at 492 nm,

*SD* standard deviation

### Application of pAbs in protection against *G. duodenalis* infection

*G. duodenalis* infected mice protected with 1/100 µg/ml pAbs showed a significant reduction in the numbers of cysts (571.11) and trophozoites (7.21) when compared to unprotected group II (4352.61 and 36.47 for cysts and trophozoites, respectively). The best result was observed with *G. duodenalis* infected group IV, protected with 1/200 µg/ml pAbs, to achieve a PR% of 99.25% and 100% for cysts and trophozoites, respectively (Table 5).

**Table 5:** parasitological examination of stool and intestinal wash samples

Groups	No. of cysts in stool samples		No. of trophozoites in intestinal wash	
	m ± SD	%PR	m ± SD	%PR
Group I	0	0	0	0
Group II	4352.61 ± 89.74	0	36.47 ± 6.9	0
Group III	571.11 ± 5.6^a^	86.87%	7.21 ± 1.8^a^	80.23
Group IV	32.24 ± 1.2^b^	99.25%	0^b^	100%

^a^represents significance when compared to group II and

^b^represents significance when compared to group III. Group I: healthy control mice, group II: *G. duodenalis* infected unprotected mice, group III: *G. duodenalis* infected mice protected with intraperitoneal injection with 100 μl of purified pAb (1/100 µg/ml) and group IV: *G. duodenalis* infected mice protected with intraperitoneal injection with 100 μl of purified pAb (1/200 µg/ml)

Levels of pro-inflammatory cytokines were highly elevated in *G. duodenalis* infected unprotected group II when compared to healthy control group I. A significant reduction in cytokines’ level was observed in *G. duodenalis* infected group IV, protected with 1/200 µg/ml pAb, when compared with group II (Table 6).

**Table 6:** levels of cytokines in serum samples of different experimental groups

Groups	IFN-γ	IL-4	IL-6
Group I	151.64 ± 3.4	13.47 ± 1.1	162.77 ± 3.4
Group II	864.44 ± 9.8	101.63 ± 4.4	654.12 ± 3.3
Group III	305.71 ± 2.2^a^	58.92 ± 5.1^a^	254.65 ± 1.7^a^
Group IV	156.41 ± 6.4^b^	19.85 ± 1.3^b^	171.32 ± 0.9^b^

^a^represents significance when compared to group II and

^b^represents significance when compared to group III. Group I: healthy control mice, group II: *G. duodenalis* infected unprotected mice, group III: *G. duodenalis* infected mice protected with intraperitoneal injection with 100 μl of purified pAb (1/100 µg/ml) and group IV: *G. duodenalis* infected mice protected with intraperitoneal injection with 100 μl of purified pAb (1/200 µg/ml)

**Fig. 1:**
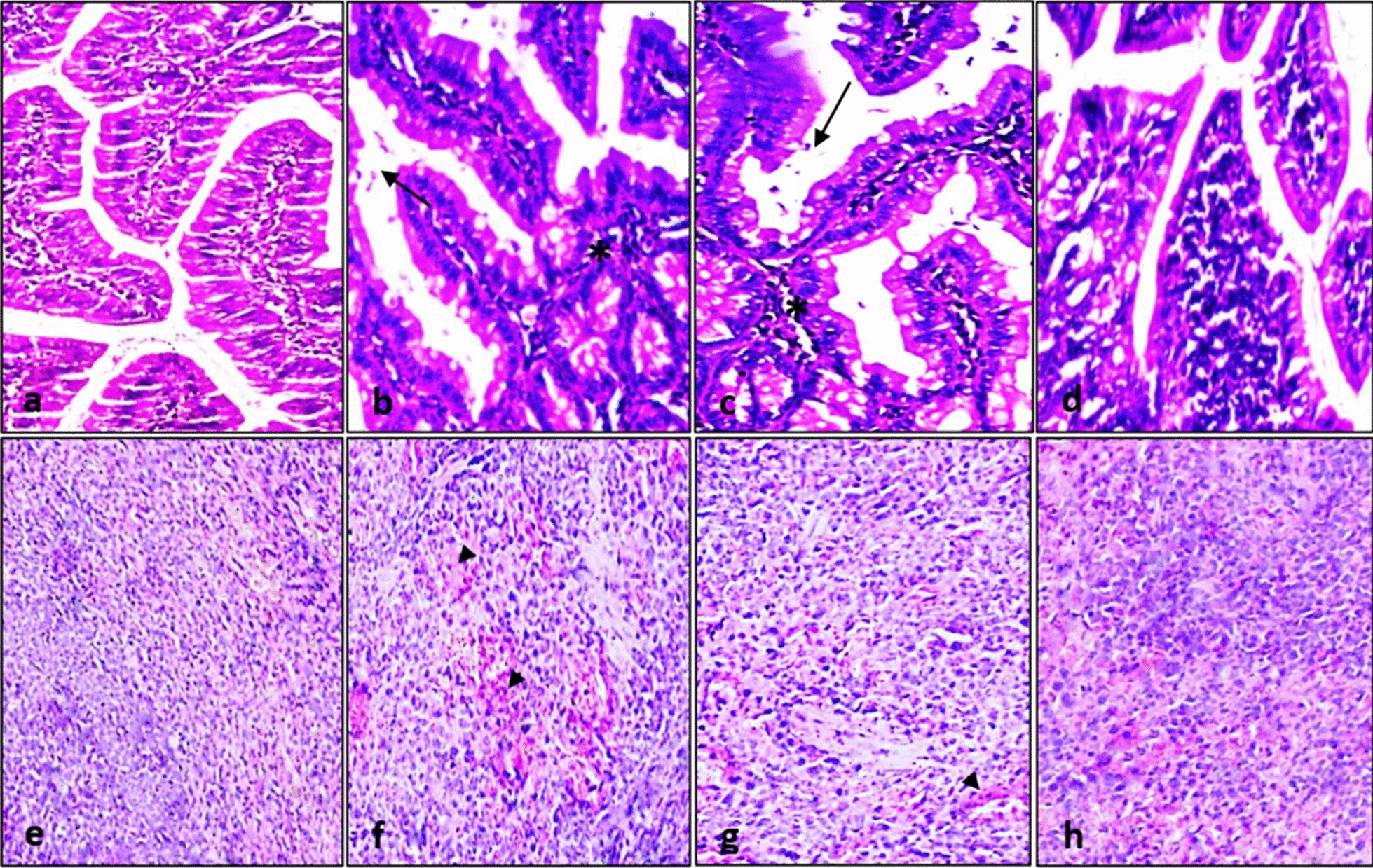
Haematoxylin and eosin intestinal sections showing preserved villous architecture and intact brush borders in healthy control group I (**a**,×200) and *G. duodenalis* infected protected group IV (1/200 µg/ml pAb, **d**, ×200), mild neutrophils infiltration (asterisks) and scattered trophozoites (arrows) in *G. duodenalis* infected unprotected group II (**b**, ×200) and *G. duodenalis* infected protected group III (1/100 µg/ml pAb, **c**, ×200). Haematoxylin and eosin spleen sections showing average lymphoid follicles and average blood sinusoids in healthy control group I (**e**, ×200) and *G. duodenalis* infected protected group IV (1/200 µg/ml pAb, **h**, ×200), average lymphoid follicles with markedly congested blood sinusoids (arrow heads) in *G. duodenalis* infected unprotected group II (**f**, ×200) and *G. duodenalis* infected protected group III (1/100 µg/ml pAb, **g**, ×200)

Healthy control group I and *G. duodenalis* infected protected group IV (1/200 µg/ml pAb) showed intestinal sections with preserved villous architecture and intact brush borders; and spleen sections with average lymphoid follicles and average blood sinusoids. On the other hand, *G. duodenalis* infected unprotected group II and *G. duodenalis* infected protected group III (1/100 µg/ml pAb) showed mild neutrophils infiltration and scattered trophozoites in intestinal sections; and an average lymphoid follicle with markedly congested blood sinusoids in spleen sections (Fig. 1).

## Discussion

*G. duodenalis* outbreaks were seen in places with insufficient chlorination facilities, inadequately managed and operated filtering devices, and even non-filtered drinking water (Daly et al. 2010). Aw et al. (2019) reported that the irregular examination of water sources increased the risk of infection by drinking polluted water; they added that the presence of *G. duodenalis* cysts in water was not affected by using albendazole. These facts were in agreement with our study; where microscopic identification of water samples reported that *G. duodenalis* cyst was found in 11 water samples. According to cyst load, 25% of water samples carry moderate cyst load and 30% have light cyst load. 15% of moderate cyst load was localized in water samples from rural areas. On the other hand, 10% of the light load was in urban areas.

Our findings were consistent with those of Ong et al. (1996) who found that lakes with livestock activities had a higher number of *G. duodenalis* cysts than those without livestock access. In addition, Odoi et al. (2004a) discovered that giardiasis prevalence was greater in rural areas and places consuming surface water, whereas rates were lower in filtered water areas and those with a high median wage. Later, in Ontario, Odoi et al. (2004b) used a variety of multivariable spatial estimation methods and discovered a statistically significant bi-variate relationship between rates of giardiasis and animal density and/or manure use in farming fields.

Discovering alternative methods for *G. duodenalis* diagnosis, rather than microscopy, had been the point of study since 2000. Fast diagnostic methods that employ antigen detection technologies have become increasingly popular in recent years. ELISA assesses soluble antigens and offers greater sensitivity compared to light microscopy (Ali et al. 1999). ELISA has also been regarded as a cost-effective diagnostic approach that can identify small amounts of parasite coproantigens, even in moderate infections, and diagnose the disease effectively even when the live parasite is not present in the stool sample (Bride et al. 1995; Yang and Harrison, 1996; Boone et al. 1999; Duque-Beltrán et al. 2002; Barazesh et al. 2010, and Barazesh et al. 2011; Silva et al. 2016; Pacheco et al. 2020). Nash and Keister (1985) categorized nineteen *G. duodenalis* isolates into three groups based on the reactivity of antibodies produced against excretory-secretory components generated in vitro in the growth media by each group. Boone et al. (1999) purified* Giardia* antigens by immunoaffinity chromatography with a monoclonal antibody (mAb). Barazesh et al. (2010) just published a quick, easy, and inexpensive approach for purifying *G. duodenalis* cysts from stool specimens of heavily infected individuals. They developed a modified technique that combines several purifications steps, including one and two-phase sucrose gradient isolation, percoll-sucrose gradient isolation, and a two-phase method that uses 0.85 and 1.5 M sucrose with certain modifications.

According to the direct smear examination method in this study, 91 patients from 100 *G. duodenalis* infected patients were positive to record sensitivity of 91%, a specificity of 100%, a PPV of 100% and a NPV of 85.93%. While according to sandwich ELISA, 97 patients were positive giving higher sensitivity (97%) than parasitological examination, a specificity of 92.72%, a PPV of 96.03% and a NPV of 94.44%. Many studies compared the enzyme immunoassay method to direct microscopic examination, such as Doruman et al. (2006) discovered that the monoclonal direct fluorescent antibody method had a sensitivity of 79.5 percent in comparison to the trichrome staining method which had a specificity of 77.8% and a sensitivity of 85.7%; and the ELISA technique which had greater sensitivity and specificity (88.6 and 88.8% respectively). El-Nahas et al. (2013) reported that ordinary microscopy identified forty of the eighty-four stool samples (47.6%) as *G. duodenalis* positive, while direct immunofluorescence and flow cytometry identified fifty-two and thirty-eight *G. duodenalis* positive cases (61.9 and 45%, respectively). Selim et al. (2009) found that giardiasis was diagnosed in forty-six patients (51.1%) by ELISA detection, but *G. duodenalis* was only found in thirty-eight patients (42.2%) by direct stool analysis. The ELISA technique achieved a 97.3% sensitivity and an 82.6% specificity, with an 80.4% PPV and a 97.7% NPV.

Worldwide waterborne giardiasis outbreaks induce *G. duodenalis* transmission to humans via contaminated water and could have a larger epidemiologic effect than previously thought. Test water for giardiasis needs the screening of thousand liters of water and a qualified examiner to check with a microscope. Even if no cysts were found, there is no certainty that they were not present in the water source. However, the US EPA 1623 method is time-consuming and complex; and when using the immunofluorescence procedure for microscopic detection, cross-reaction with non-target organism like algae is at a severe disadvantage. Furthermore, Fluorescein isothiocyanate (FITC) conjugated mAb have different cross-reactivity degrees with protozoan cysts. Alternative solutions have been suggested, but they require a lot of centrifuging and highly-priced chemicals to detect DNA molecularly.

In our study, ordinary microscopic identification of protozoa stages in water samples (n = 20), *Giardia* cyst was found in seven water samples. On the other hand, when sandwich ELISA was used for detection using IgG pAb; nineteen water samples gave positive results. The sandwich ELISA's remarkable efficiency is due to its capacity to bind surface antigens without compromising the *G. duodenalis* cyst integrity; and identifying modest levels of antigens, indicating a low parasite load. For surveys of *G. duodenalis*, *E. histolytica*, and *Cryptosporidium sp.* in water and soil samples, Da Silva et al*.* (2012) compared the similarity between Sheather's technique modified by Huber, Ritchie's technique modified by Young, and the ELISA method. When compared to microscopic approaches, ELISA revealed an increased number of positive samples examined, both in water and soil. Furthermore, when ELISA is compared to direct immunofluorescence, which uses mAb with great sensitivity and is capable of detecting undivided cysts and oocysts, ELISA has the advantages of being less expensive, faster, and easier to handle. Other important considerations include the requirement for less technological infrastructure, as certain kits may be processed visually and do not require a qualified technician to identify evolving parasite species.

Passive immunization with a particular pAbs and/or mAbs protects mice from infections (DuBourdieu, 2019). After a long period of neglect, there is renewed interest in investigating antibody-based immunity in order to completely recognize the immune response to infections (Guirado et al. 2006). Selim et al. (2017) reported the protective effect of anti-*G. duodenalis* antibodies in chicken egg yolk. The author tested the prepared antibodies in vivo and demonstrated its protective effect. The present study demonstrated that 1/200 µg/ml of pAbs protected mice from giardiasis; this was evident from the reduction in cysts number in the stool. The histopathological examination of the intestinal sections revealed the absence of trophozoites.

In conclusion, due to the irregular elimination of *G. duodenalis* cysts in stool, microscopic detection has poor sensitivity and needs to be performed by qualified persons for an accurate assessment. For the purpose of identifying parasite antigens in stool, coproantigen tests based on ELISA are more accurate than those that use microscopy. Also, the prepared IgG pAbs succeeded in the diagnosis of *G. duodenalis* antigens in both water and stool samples. Moreover, our results recommended the use of sandwich ELISA as a diagnostic technique. The pAbs can be prepared in a large amount, used in field diagnosis and protection. This will help in preventing giardiasis outbreaks in rural areas. Also, to our knowledge, this is the first study which reported the protective effect of anti-*G. duodenalis* pAbs in vivo.

## Data Availability

All data generated or analysed during this study are included in this published article.
